# G-C_3_N_4_-Orchestrated Multi-Component Synergy for Enhanced Flame Retardancy in PLA

**DOI:** 10.3390/polym18212622

**Published:** 2026-10-23

**Authors:** Yu-Ting Kuo, Jazmine Aiya D. Marquez, Wan Zhang, Zihao Wang, Qingsheng Wang

**Affiliations:** Artie McFerrin Department of Chemical Engineering, Texas A&M University, College Station, TX 77843, USA

**Keywords:** polylactic acid, flame retardancy, pre-assembly strategy, graphitic carbon nitride, fumed silica, ammonium polyphosphate, lignin

## Abstract

Polylactic acid (PLA) is a biodegradable polymer with good processability, but its poor flame retardancy limits its wider practical use. In this work, an ethanol-assisted pre-assembled g-C_3_N_4_/fumed silica hybrid (gCNFS) was introduced into a PLA/ammonium polyphosphate (APP)/lignin system to improve its flame-retardant performance. The pre-assembly strategy was designed to improve the interfacial contact between g-C_3_N_4_ and fumed silica and regulate char formation and thermal decomposition during burning. The chemical structure and morphology of the composites were analyzed to study their thermal degradation behavior, volatile release, and post-burn char morphology. The results showed that the introduction of pre-assembled gCNFS improved the flame retardancy of the PLA/APP/lignin system. Among all formulations, PLA/Q-gCNFS-3 exhibited the best overall performance, achieving a limiting oxygen index (LOI) of 37% and a UL-94 V-0 rating. Compared with neat PLA, its peak heat release rate (pHRR) and total heat release (THR) decreased by approximately 50% and 24%, respectively, indicating effective suppression of burning intensity and total heat output. The flame-retardant mechanism of PLA/APP/Lignin with gCNFS enhanced both condensed-phase and gas-phase flame-retardant actions. In the condensed phase, a continuous and compact expanded char layer with better sealing ability and barrier performance was formed. In the gas phase, nonflammable gas release occurred, which helped reduce the concentration of combustible volatiles. Hence, the ethanol-assisted pre-assembled gCNFS is an effective strategy for improving the flame-retardant efficiency of PLA/APP/lignin composites.

## 1. Introduction

Polylactic acid (PLA) is a bio-based and degradable thermoplastic polyester made using a polycondensation reaction or ring-opening polymerization (ROP) of lactic acid [1,2]. It is widely used in food packaging [3], disposable tableware [4], and 3D printing [4]. At the same time, its biocompatibility enables applications in the medical field, such as absorbable sutures [5], bone fixation devices [6], and tissue-engineered stents [7]. It has also expanded to automotive [8] and electronic [9] applications. Its relatively low carbon emissions [10,11] and ability to decompose under industrial composting conditions [12,13] are also attractive advantages. Moreover, it can also be processed through traditional thermoplastic techniques such as melt extrusion, injection molding, film blowing [14], and 3D printing [4]. However, PLA’s high flammability limits its application.

Biomass-based flame retardants have received increasing attention because they are renewable and can support char formation during polymer combustion. A common design strategy is to combine carbon-, nitrogen-, and phosphorus-containing components, where carbon-rich structures help form protective char, phosphorus species promote dehydration and char formation, and nitrogen-containing species release nonflammable gases to dilute combustible volatiles [15]. Based on this concept, the APP/lignin flame-retardant system has been developed as an intumescent flame-retardant (IFR) strategy for PLA. APP is a halogen-free flame retardant with high phosphorus content and several ammonium groups, which can act as both an acid source and a gas source [16]. On the other hand, lignin is an aromatic polymer with high carbon content and can serve as a carbon-rich precursor for char formation [17]. This system is commonly described as a P–N–C synergy. During burning, APP generates phosphorus-containing radicals (e.g., PO·) that intercept H· and OH·, weakening the gas-phase chain reaction, and releases nitrogen-containing non-combustible gases (e.g., NH_3_) that dilute the flame zone [18,19]. In parallel, lignin provides a carbon-rich structure for building an expanded protective char layer [20,21]. This layer blocks heat and oxygen, while limiting the release of flammable volatiles [22,23,24,25]. The effectiveness of the APP/lignin FR system in PLA was demonstrated in several studies. Zhu et al. developed a bio-based core–shell flame retardant by coating APP with chitosan and lignin (APP@CS@LK), and the PLA composite with 10 wt% loading achieved UL-94 V-0, increased the LOI from 19.5% to 30.7%, and reduced pHRR by 23.5% [25]. Peil et al. reported that APP-loaded lignin nanocontainers improved the dispersion of APP in PLA foam and helped the system reach UL-94 V-0, with the LOI increasing from 21.0% to 24.5% [20]. Yoo et al. also showed that combining APP with phenolated and silylated lignin improved flame retardancy and produced a more cohesive residue layer in PLA [26]. However, despite these improvements, the intumescent char formed in PLA/APP/lignin systems is often still weak, porous, and prone to cracking, which limits its barrier effect under sustained heating.

To address these limitations, many studies have introduced inorganic additives (e.g., SiO_2_ or clays) or used Si/P-type lignin modifications to increase the char density and toughen the residue [26,27]. However, dispersion and interfacial compatibility remain important issues because APP is relatively hydrophilic, PLA is hydrophobic, and lignin is structurally heterogeneous [20,28]. As a result, localized phase separation can still occur at high loadings [20,29,30,31], which may disrupt char layer integrity and lead to non-uniform protective layers during burning. In addition, the balance between condensed-phase protection and gas-phase suppression is not easy to optimize. Some PLA flame-retardant systems can form char, but their overall fire performance still depends on whether gas-phase dilution and condensed-phase protection are well balanced [32]. Therefore, prior studies suggest that an improved PLA/APP/lignin design should build a tougher and more continuous char barrier to achieve high flame-retardant efficiency at lower additive loadings.

Graphitic carbon nitride (g-C_3_N_4_, gCN) is a layered structure that can act as a physical barrier during burning [33]. It can slow down heat and mass transfer and delay the escape of flammable volatiles into the pyrolysis zone [34,35]. It may also support the intumescent char, making the char more continuous, which reduces crack formation under sustained heat [34]. In addition, the nitrogen-ric structure of gCN may promote the formation of P–N–C structures with APP-derived species, while phosphoric species from APP can react with oxygen-containing groups in lignin to form P–O–C structures. These structures help produce a denser and more oxidation-resistant char [36]. During thermal decomposition, APP is the main source of nonflammable gases such as NH_3_. The nitrogen-rich gCN framework may further support the APP-related flame-retardant pathway, but its main role is still considered to be the reinforcement of the condensed-phase barrier [33,37]. However, whether these potential benefits can consistently translate into flame-retardant performance depends strongly on maintaining an intact, well-contacted hybrid structure. If severe restacking of 2D nanosheets occurs, it can create defect sites in the char layer that weaken barrier continuity and promote cracking [38,39].

Based on this, fumed silica (FS) is used to prevent the restacking of gCN nanosheets. FS has a high specific surface area and abundant surface silanol groups. It can readily form particle–particle contact networks in composites that serve as “spacers” and support points between gCN layers, reducing the tendency for restacking [35,40,41]. More importantly, in intumescent systems, inorganic SiO_2_ phases are often used to improve the structural stability and compactness of the char layer [42,43]. However, despite reported gCN/APP synergies [33,37] and silica-assisted char strengthening [42,43], simple physical dry blending of gCN and FS fails to resolve the dispersion bottleneck, as 0D FS nanoparticles are also prone to self-aggregation and cannot establish uniform interfacial contacts with 2D gCN under high-shear melt compounding. To overcome this limitation, an ethanol-assisted liquid-phase pre-assembly strategy is introduced to regulate the contact between solid additives prior to melt blending. In ethanol suspension, ultrasonic processing facilitates non-covalent hydrogen bonding and physical adsorption between FS silanol groups and gCN nitrogen sites. This pre-assembled 0D/2D hybrid architecture effectively prevents gCN restacking while suppressing FS self-aggregation during extrusion, enabling a controlled comparison with direct gCN addition. Thus, this study aims to clarify what FS-driven pre-assembly adds beyond conventional multi-component physical blending in the PLA/APP/lignin IFR system. Stable gCN–FS contacts are pre-built in a solvent and then introduced into the composite, enabling a controlled comparison with direct gCN addition. Flammability tests (MCC, cone, LOI, UL-94) with thermal and chemical analyses (TGA/TG-MS, FTIR) and char morphology observations (macroscopic photos, SEM) are combined to connect structure with decomposition and burning behavior. Based on this evidence, this study clarifies how the pre-assembled 0D/2D hybrid architecture and interfacial interactions govern char quality, dripping/self-extinguishing behavior, and overall flame-retardant efficiency.

## 2. Materials and Methods

### 2.1. Materials

Polylactic acid (PLA, Ingeo™ Biopolymer 4032D) was purchased from NatureWorks LLC (Minnetonka, MN, USA). Ammonium polyphosphate (APP, AFLAMMIT PCI 202) was supplied by Thor Specialties, Inc. (Seyer, Germany). Fumed silica powder with an aggregate size of 0.2–0.3 μm and Melamine (C_3_H_6_N_6_, 99%) were obtained from Sigma Aldrich (St. Louis, MO, USA). Alkaline lignin was supplied by Tokyo Chemical Industry Co, Ltd. (Tokyo, Japan), while ethanol (200 proof) was obtained from Reagent Inc. (Charlotte, NC, USA).

### 2.2. Composite Preparation

#### 2.2.1. G-C_3_N_4_ Preparation

**Figure 1:**
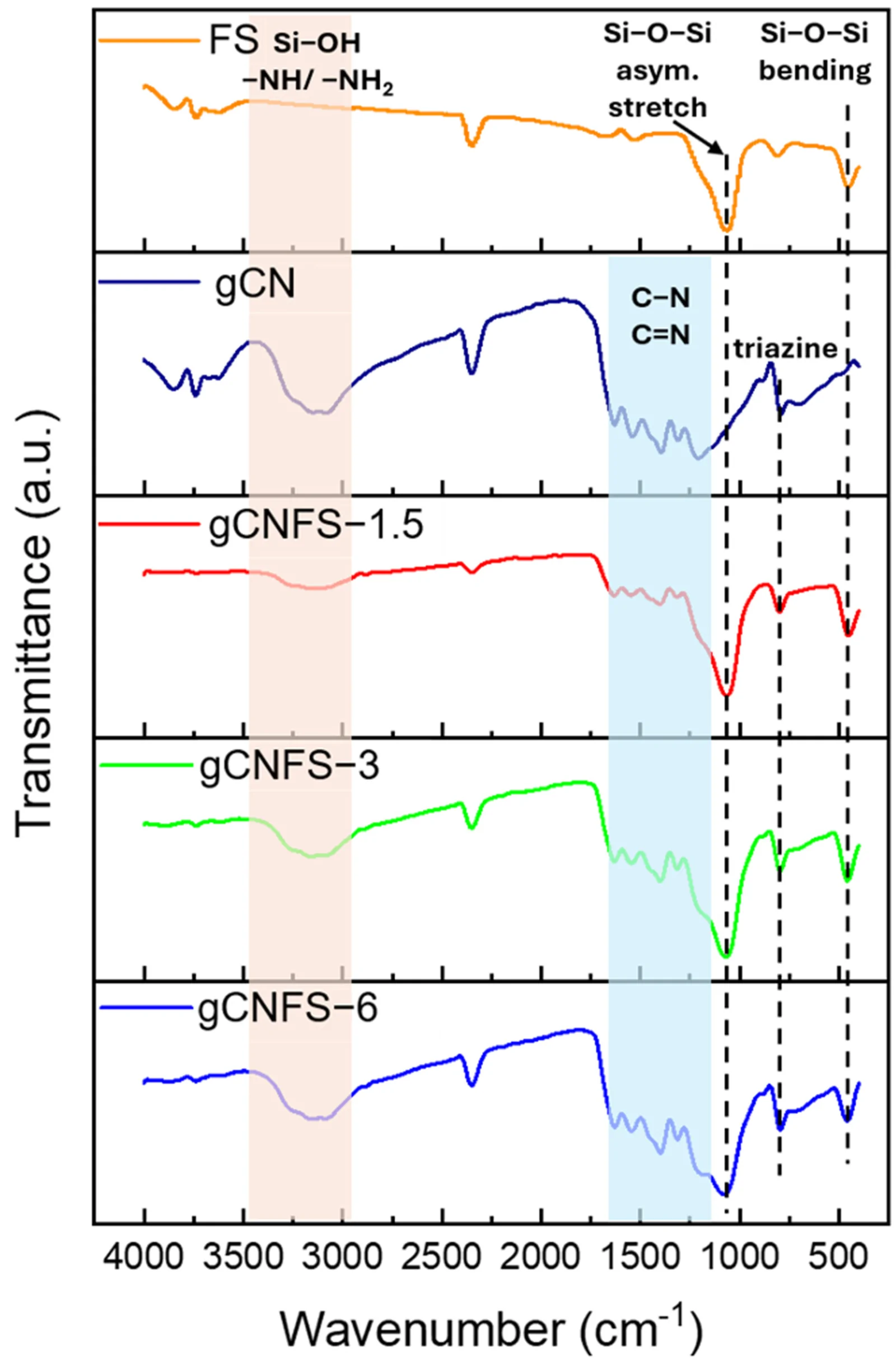
FTIR spectra of FS-gCN pre-assembly samples.

The synthesis of g-C_3_N_4_ powder was conducted via thermal condensation of the melamine precursor. The sample was heated in air at a rate of 4.5 °C/min to 550 °C in a muffle furnace; the temperature was held for 4 h, and then naturally cooled to room temperature. The typical mass yield of g-C_3_N_4_ was approximately 45%. The successful polycondensation and structural purity of g-C_3_N_4_ were confirmed by FTIR spectroscopy (Figure 1), which showed characteristic heptazine ring vibrations without residual melamine monomer peaks. The obtained light-yellow product was ground into fine powder, collected, and stored for further use.

#### 2.2.2. gCNFS Preparation

First, g-C_3_N_4_ and fumed silica were mixed in a 95% ethanol (*v*/*v*) aqueous solution (prepared by diluting 200 proof ethanol with deionized water) according to the formulations listed in Table 1. The suspension was dispersed using a probe-type sonicator (Branson Sonifier 250, Branson Ultrasonics, Danbury, CT, USA) at an output setting of 6 with a duty cycle of 40% for 20 min in pulse mode to minimize overheating. Under this fixed condition, the FS level was treated as the primary variable in the pre-assembled powders. The suspension was then dried in a vacuum oven at 70 °C for 12 h. The powder obtained after drying and grinding is the gCNFS sample with different addition ratios. To eliminate processing variables related to solvent exposure and ultrasonic energy, the baseline PLA/Q-gCN sample was prepared using pristine g-C_3_N_4_ powder that was subjected to the exact same ethanol suspension, ultrasonic probe treatment (20 min in pulse mode), and vacuum drying procedures as those used for the gCNFS hybrids. In this study, fumed silica (FS) was specifically employed as a nano-spacer to regulate gCN dispersion through a liquid-phase pre-assembly route, rather than as an independent bulk additive. Therefore, the PLA/Q-gCN formulation serves as the primary baseline to systematically evaluate the synergistic role and optimal ratio of the pre-assembled 0D/2D gCNFS hybrid.

#### 2.2.3. Preparation of Standardized Composite PLA Material Specimens

Before melting and compounding, PLA pellets, APP, lignin, and gCN or the pre-assembled gCNFS additives were dried in a vacuum oven at 80 °C for 6 h to remove residual moisture. Except for neat PLA (100 wt% PLA), all flame-retardant composites contained 75 wt% PLA, 15 wt% APP, and 7 wt% lignin, together with 3 wt% of either gCN (PLA/Q-gCN) or the corresponding pre-assembled gCNFS hybrid powder (PLA/Q-gCNFS-1.5, PLA/Q-gCNFS-3, and PLA/Q-gCNFS-6). The melt compounding was then carried out following the formulations in Table 1 to obtain PLA/Q-gCN, PLA/Q-gCNFS-1.5, PLA/Q-gCNFS-3, and PLA/Q-gCNFS-6. To provide full clarity on the chemical formulation, the designation ‘Q’ in the sample codes represents the quaternary flame-retardant system consisting of 75 wt% PLA, 15 wt% APP, 7 wt% lignin, and 3 wt% of the fourth additive component (g-C_3_N_4_ or gCNFS hybrids). As detailed in Table 1, the absolute loading of fumed silica (FS) in the final PLA/Q-gCNFS composites is strictly regulated at 1.20 wt%, 0.75 wt%, and 0.43 wt% for PLA/Q-gCNFS-1.5, PLA/Q-gCNFS-3, and PLA/Q-gCNFS-6, respectively, while maintaining a constant total quaternary additive dosage of 3.0 wt%. The composites were prepared and extruded using a twin-screw extruder (Process 11 co-rotating parallel twin-screw extruder, Thermo Fisher Scientific, Waltham, MA , USA) [44]. The screw diameter was 11 mm, and the length-to-diameter ratio (L/D) was 40. The materials were fed using a gravity feeder with a 12 mm screw diameter and an L/D of 8. During melt compounding, the barrel temperature profile of the twin-screw extruder was set to 160 °C, 160 °C, 180 °C, 180 °C, 185 °C, 185 °C, 185 °C, and 185 °C, and the screw speed was 60 rpm. After extrusion, all samples were pelletized and then compression-molded using a standard hot press (Carver Inc., Wabash, IN, USA; Model 4120) at 175 °C under a load of 6 tons for 30 min. The samples were then cooled naturally to room temperature under the same load. Finally, specimens with a thickness of 3.2 mm were obtained and cut into standard shapes according to the corresponding test standards. To evaluate the baseline performance of the ternary intumescent flame-retardant (IFR) matrix without quaternary additives, a reference sample containing PLA, APP, and lignin (PLA/APP/Lignin, 75/17/8 *w*/*w*/*w*) was also tested, with its MCC parameters provided in Table S1 (Supplementary Material).

### 2.3. Fire Testing Methods

Microscale combustion calorimetry (MCC) (Fire Testing Technology, East Grinstead, UK) was conducted using a pyrolysis–combustion flow calorimetry setup in triplicate (N = 3). About 4 mg of material was heated from 150 to 600 °C at a heating rate of 1 °C/s in a nitrogen environment, where the sample was pyrolyzed in 80 cm^3^/min of flowing nitrogen gas to simulate the anaerobic conditions beneath a flame. The evolved gases are then mixed with a controlled oxygen stream at a rate of 20 cm^3^/min in the combustor, and heat release is quantified based on the oxygen consumption principle. The collected MCC data were processed using MCC Curve Fit 17 software (Fire Testing Technology Ltd., East Grinstead, UK). Limiting Oxygen Index (LOI) was measured on an FTT oxygen index apparatus (Fire Testing Technology, UK) in accordance with ASTM D2863 with dimensions of 125 mm × 6.5 mm × 3.2 mm. UL-94 vertical burning behavior was evaluated following ASTM D3801 using a horizontal/vertical flame chamber (Fire Testing Technology Ltd., East Grinstead, UK). UL-94 specimen dimensions used were 130 mm × 13.0 mm × 3.2 mm, with 5 replicate specimens (N = 5). Cone calorimetry was used as a bench-scale method to assess reaction-to-fire behavior under well-ventilated, forced-combustion conditions. Tests were carried out according to ASTM E1354 using an iCone classic calorimeter (Fire Testing Technology, UK) with 5 replicate specimens (N = 5). All samples were 100 mm × 100 mm × 3.2 mm. A spark ignitor was applied to achieve sustained ignition, and the external heat flux was set to 50 kW/m^2^ to represent a severe fire exposure. Due to raw material and sample supply limitations, Cone calorimetry tests were conducted as standard single-run screening evaluations (N = 1) under strictly controlled testing conditions.

All quantitative fire test parameters measured with multiple runs (LOI, UL-94 after-flame times, and MCC metrics) are reported as Mean ± Standard Deviation (SD).

### 2.4. Characterization

ATR-FTIR was used to analyze the functional groups of additives and combustion residues using a Thermo Scientific Nicolet iS5 spectrometer with an iD7 ATR attachment (Waltham, MA, USA). The spectra were recorded in the wavenumber range of 4500–0 cm^−1^ at a resolution of 4 cm^−1^ over 50 scans. Thermal stability of neat PLA and PLA composites was assessed under a thermo-oxidative atmosphere (air atmosphere with a sample gas flow rate of 50 mL/min and a balance nitrogen flow rate of 40 mL/min) using a TGA Q50 (TA Instruments, New Castle, DE, USA), with samples heated from 30 to 700 °C at a ramp rate of 10 °C/min. For TG-MS analysis, a fixed initial sample mass (~4.0 mg) was pyrolyzed for each run under identical gas flow conditions to enable reliable qualitative and comparative evaluation of evolved volatiles. The TGA outlet was connected via a capillary line to a Stanford Research Systems UGA200 Universal Gas Analyzer (Sunnyvale, CA, USA) equipped with a mass spectrometer to monitor evolved gases during heating; the MS detects volatile fragments based on their mass-to-charge (*m*/*z*) ratios, allowing temperature-resolved interpretation of major gas products associated with each mass loss stage. The morphology of char residues after burning was examined using a SNE-ALPHA tabletop SEM system. SEM images were collected at an accelerating voltage of 10 kV with a magnification of 100.

## 3. Results and Discussion

### 3.1. Pre-Assembly and Chemical Signatures

#### 3.1.1. FTIR Spectra of gCNFS

Figure 1 shows the FTIR spectra of pristine FS, gCN, and the FS-gCN pre-assembly samples. For pristine gCN, the spectrum exhibits three diagnostic regions: the sharp band at 810 cm^−1^ corresponding to the out-of-plane bending vibration of tri-s-triazine (heptazine) units, the intense bands in the 1200–1650 cm^−1^ region belonging to aromatic C–N and C=N heterocycles, and a broad envelope centered at 3000–3300 cm^−1^ assigned to terminal –NH/–NH_2_ groups. Notably, the complete absence of sharp –NH_2_ stretching doublets at 3400–3500 cm^−1^ (characteristic of melamine) confirms the complete polycondensation of melamine into pure gCN without unreacted monomer contamination. FTIR analysis shows that the pre-assembled samples retain both the characteristic Si–O–Si absorption bands of FS and the triazine/heptazine framework bands of gCN, indicating that both components remain present after pre-assembly rather than one phase being lost or structurally destroyed. Compared with pristine FS and gCN, the pre-assembled samples display a distinct blue-shift of the characteristic Si–O–Si asymmetric stretching vibration band to higher wavenumbers—shifting from 1070.0 cm^−1^ in pristine FS to 1076.3 cm^−1^ for gCNFS-1.5, 1075.5 cm^−1^ for gCNFS-3, and 1079.9 cm^−1^ for gCNFS-6—with a substantial maximum shift of up to 9.9 cm^−1^. Meanwhile, noticeable band broadening is observed in the 1200–1650 cm^−1^ region assigned to the nitrogen-containing framework of gCN. Furthermore, a broad absorption band persists in the 3200–3500 cm^−1^ region across all hybrid samples. This region arises from the overlapped contributions of surface Si–OH groups, terminal –NH/–NH_2_ groups on gCN, and adsorbed water. After pre-assembly, this broad band becomes less resolved than that of pristine gCN, indicating that these OH/NH-related groups are redistributed into more complex hydrogen-bonding environments [37]. These spectral changes suggest that FS and gCN do not simply undergo mechanical mixing but instead form interfacial interactions that modify the chemical environment on both surfaces. Based on the changes in the broad OH/NH region, the Si–O–Si band, and the gCN framework region, the interaction is more reasonably attributed to hydrogen bonding and surface adsorption between silanol groups on FS and nitrogen-containing sites on gCN, rather than the formation of a clearly defined new covalent bond. This interpretation is consistent with studies showing that silica and g-C_3_N_4_ can form interfacial interactions through surface silanol groups and nitrogen-containing sites [45]. Therefore, the observed peak shifts, band broadening, and reduced shoulder resolution can be attributed to hydrogen-bond-driven interfacial assembly and surface adsorption between FS and gCN, rather than simple physical blending [39].

#### 3.1.2. FTIR Spectra of PLA/FR Composites

**Figure 2:**
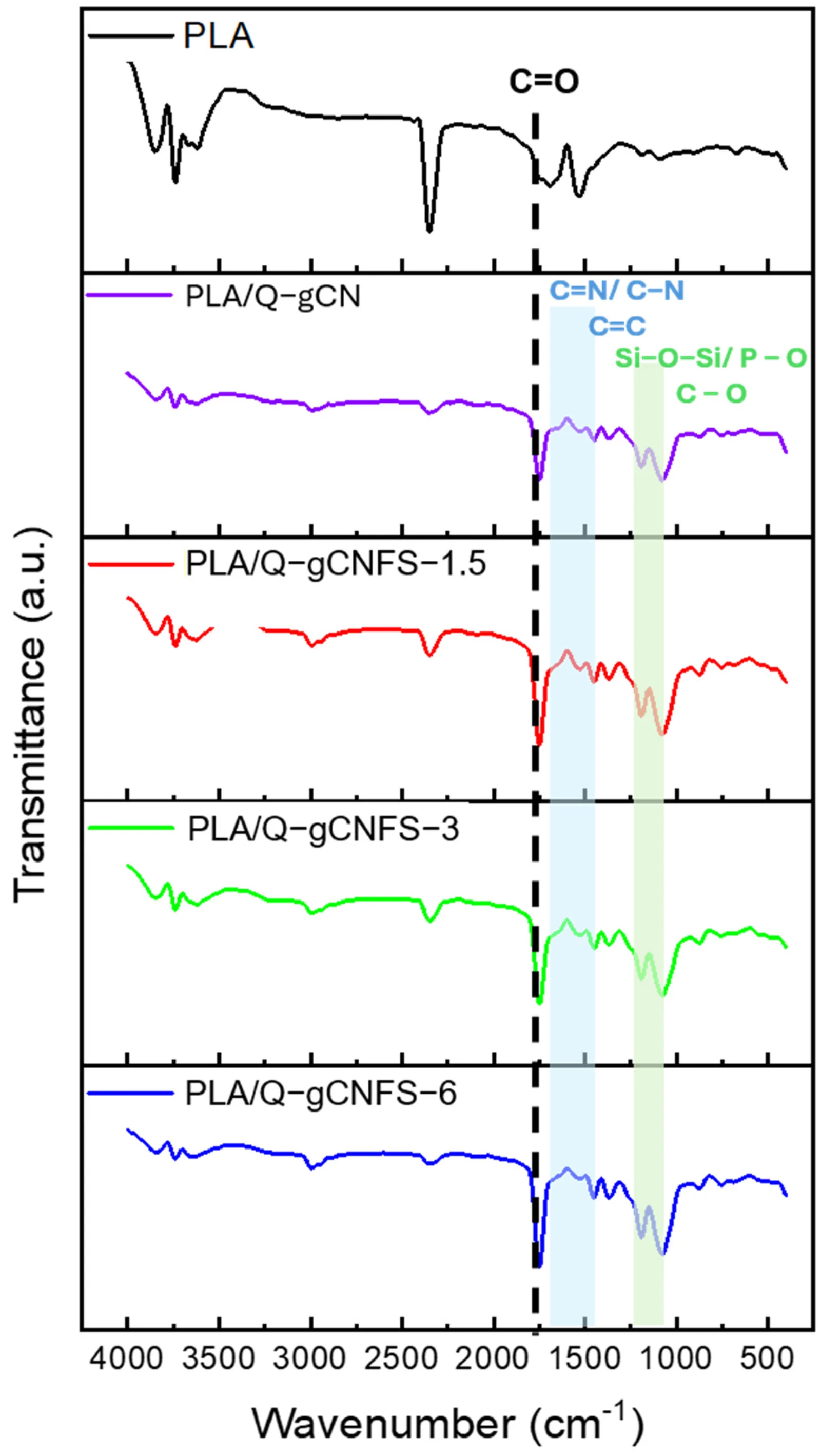
FTIR spectra of PLA/FR Composites.

Figure 2 shows changes in the FTIR band positions of the composites containing pre-assembled Q-gCNFS compared with PLA/Q-gCN. The ester C=O absorption band of PLA remains around 1751 cm^−1^ after the introduction of Q-gCNFS, with minimal variation in peak position. This indicates that the main PLA ester structure is retained, while changes in band location suggest modified chain packing and intermolecular interactions within the composite. In addition, the absorption features in the 1650–1500 cm^−1^ region become more distinct in the Q-gCNFS-containing samples, and the overlapped bands in the 1180–1000 cm^−1^ region are also strengthened. These changes indicate that the interfacial interactions in the Q-gCNFS series are different from those in PLA/Q-gCN, which is attributed to the presence of the pre-assembled gCNFS hybrid. Such changes are consistent with previous studies showing that variations in FTIR band shape and position in PLA-based systems are related to differences in intermolecular interactions and interfacial compatibility [46,47]. Therefore, the results suggest that the interfacial interactions generated during pre-assembly are retained in the composite, mainly through weak intermolecular interactions—specifically hydrogen bonding (e.g., between the surface silanol Si–OH/amine –NH_2_/–NH groups of gCNFS and the carbonyl C=O/ester oxygen C–O–C groups of PLA) and dipole–dipole interactions—as well as physical surface adsorption, rather than the formation of new covalent bonds.

### 3.2. Thermal Stability (TGA/DTG)

**Figure 3:**
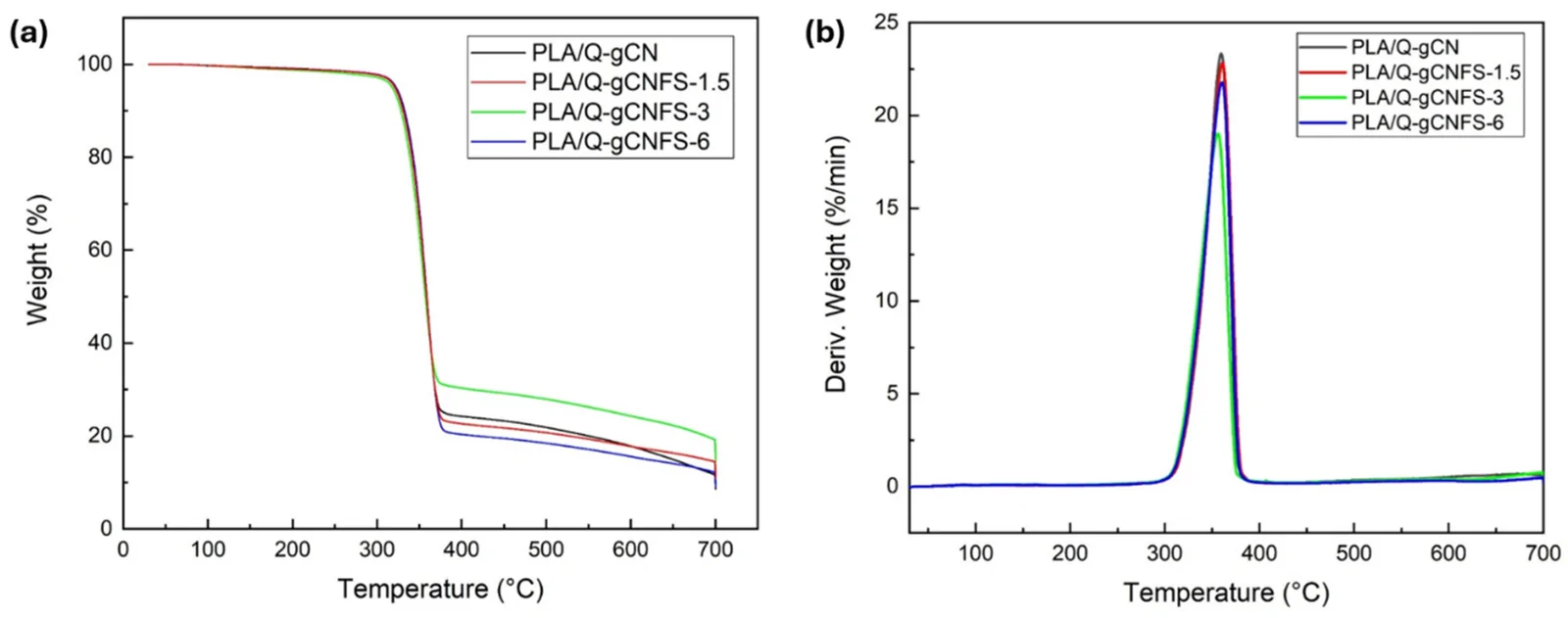
TGA (**a**) and DTG (**b**) curves of PLA/FR composites.

Thermal stability was evaluated to understand the thermal decomposition behavior of the polymer composite. In this process, the release of combustible volatiles and the formation of protective char could be identified, both of which are closely related to fire behavior. To explicitly clarify the thermal decomposition behavior of the composites, the benchmark TGA/DTG metrics of individual pristine components—neat PLA, pure APP, and pure lignin—were cited from representative literature for direct comparison [48,49,50]. Neat PLA exhibits a severe single-stage pyrolysis with an onset temperature (T_5%_) of ~330 °C and a maximum degradation rate temperature (T_max_) of ~368 °C, leaving almost zero residue at 700 °C (<1.5 wt%) [48]. Pure APP degrades through a two-step process starting earlier (T_5%_ ≈ 295 °C, T_max1_ ≈ 335 °C), releasing NH_3_ and H_2_O to yield crosslinked polyphosphoric acid with~18.5 wt% residue at 700 °C [49]. Pristine lignin demonstrates a broad thermal degradation spectrum from 200 to 600 °C (T_5%_ ≈ 220 °C, T_max_ ≈ 360 °C) due to continuous aromatic ring condensation, achieving a high char residue of ~38.0 wt% at 700 °C [50]. As shown in Figure 3a,b, the primary degradation stage of all PLA/Q-gCNFS composites occurs around 320–330 °C, which represents the overlapped pyrolysis of PLA, APP, and lignin. Although the early decomposition of APP and lignin slightly lowers the T_5%_ of the composites compared to pure PLA, it induces premature dehydration and charring of PLA chains. Consequently, the maximum degradation rate (R_max_ decreases from 23.3%/min (PLA/Q-gCN) down to 19.0%/min (PLA/Q-gCNFS-3), while the high-temperature residue at 700 °C increases substantially to 14.7% (compared to <1.5% for neat PLA). This proves that the combination of APP, lignin, and gCNFS effectively transitions the pyrolysis process from complete volatile degradation toward stable protective char formation [46,51,52]. The onset degradation temperatures (T_5%_, corresponding to 95% mass retention) are 323, 321, 318, and 322 °C for PLA/Q-gCN, PLA/Q-gCNFS-1.5, PLA/Q-gCNFS-3, and PLA/Q-gCNFS-6, respectively. These minor fluctuations in T_95%_ (within 318–323 °C) fall within normal experimental margin of error, indicating that the initial thermal stability remains statistically comparable across all composite formulations. Although PLA/Q-gCNFS-3 shows an earlier initial mass loss, it gives the highest residue at 700 °C, reaching 14.7%, compared with 8.6%, 11.2%, and 9.0% for PLA/Q-gCN, PLA/Q-gCNFS-1.5, and PLA/Q-gCNFS-6, respectively. This result indicates that PLA/Q-gCNFS-3 does not simply delay decomposition, but more effectively promotes residue formation during heating.

The DTG curves in Figure 3b further show that all samples have one dominant degradation peak, all occurring between 362–368 °C, indicating that PLA decomposition and the degradation of the flame-retardant components occur within the same temperature range. The maximum degradation rates are 23.3, 21.8, 19.0, and 22.8% min^−1^ for PLA/Q-gCN, PLA/Q-gCNFS-1.5, PLA/Q-gCNFS-3, and PLA/Q-gCNFS-6, respectively. Overall, among the tested composites, PLA/Q-gCNFS-3 shows the lowest degradation rate and the highest final residue, indicating the most favorable thermal decomposition behavior.

### 3.3. Fire Testing and Combustion Analysis

#### 3.3.1. Micro-Scale Combustion Calorimetry (MCC)

Micro-scale combustion calorimetry (MCC) was used to evaluate the intrinsic heat release behavior of the materials during controlled thermal decomposition. This method helps determine how much heat is released from pyrolysis gases at the microscale, which is closely related to the flammability potential of the composites.

Table 2 presents the MCC test of PLA and PLA/FR composites. Pure PLA shows the highest heat release among the tested samples, with a pHRR of 449 W/g and a THR of 16.5 kJ/g. This strong heat-release response is associated with the rapid generation and combustion of volatile products during decomposition. After introducing APP/lignin/gCN/FS, all formulations show reduced heat release. PLA/Q-gCNFS-3 and PLA/Q-gCNFS-6 exhibit lower pHRR values of 334 and 324 W/g, respectively, with THR values of 12.6 and 12.0 kJ/g. Compared with the ternary PLA/APP/Lignin baseline (Table S1, pHRR = 415.3 W/g, THR = 12.9 kJ/g, char yield = 12.9 wt%), the incorporation of 3 wt% Q-gCN or Q-gCNFS hybrids leads to a noticeable reduction in both pHRR and THR, alongside an increase in char residue. This comparison confirms that adding gCN and pre-assembled gCNFS hybrids effectively enhances the flame-retardant efficiency of the primary PLA/APP/Lignin matrix. The reduction in heat release is related to changes in the decomposition pathway. APP can promote dehydration reactions, while lignin provides aromatic structures that favor carbonization rather than complete volatilization. Meanwhile, gCN and FS can act as thermally stable surfaces during pyrolysis. These surfaces restrict the direct escape of degradation fragments, increase contact between decomposition products and flame-retardant components, and help shift part of the degradation products from combustible volatiles toward less heat-releasing products. As a result, both the rate and the total amount of heat release are reduced.

The T_max_ values remain within a narrow range of 384–388 °C, compared with 392 °C for pure PLA. This suggests that the temperature at which the pHRR was achieved has not strongly shifted, while the HRR is clearly reduced. In other words, the flame-retardant components do not mainly delay the main decomposition peak. Instead, they reduce the amount and/or combustibility of volatile products released in this region and promote the formation of residue. It is worth noting that the performance rankings of formulations in MCC differ slightly from those observed in bench-scale fire tests. Specifically, PLA/Q-gCNFS-6 exhibited the lowest pHRR (324.0 W/g) and highest char yield (18.2%), whereas PLA/Q-gCN achieved the lowest THR (11.5 kJ/g), and PLA/Q-gCNFS-1.5 showed higher heat release than PLA/Q-gCN. These trends are governed by the microscale nature of the MCC test, which pyrolyzes milligram-scale samples (~4 mg) without forming a macro-scale protective char barrier. In PLA/Q-gCNFS-6, the highest absolute g-C_3_N_4_ loading (2.57 wt%) maximizes intrinsic thermal stability and nitrogenous residue formation during pyrolysis. Conversely, in PLA/Q-gCNFS-1.5, the higher FS fraction (1.20 wt%) dilutes the active g-C_3_N_4_/APP/lignin flame-retardant network without contributing to physical barrier formation in a pyrolysis cup. Overall, the MCC results indicate that the flame-retardant system reduces the generation and combustion of volatile fuels at the microscale. However, MCC is conducted under a controlled environment and does not directly describe flame spread or self-extinguishing behavior, so LOI and UL-94 tests are needed to further evaluate the burning behavior of these composites.

#### 3.3.2. Inherent Fire Properties (UL-94, LOI)

Limiting oxygen index (LOI) and UL-94 vertical burning tests were used to evaluate the inherent flame-retardant behavior of the materials. Specifically, UL-94 reflects flame spread, dripping behavior, and self-extinguishing ability under direct flame exposure, whereas LOI measures the minimum oxygen concentration required to sustain combustion [53].

As shown in Table 3, pure PLA shows rapid softening and dripping during UL-94 testing, and the burning droplets ignite the cotton, resulting in a V-2 rating. In contrast, all flame-retardant composites achieve a V-0 rating. Although physical melt dripping is still observed across all flame-retardant composite formulations (marked as “Y” in Table 3), the nature of the dripping is fundamentally altered compared to neat PLA. Neat PLA exhibits severe flaming dripping that readily ignites the surgical cotton (V-2 rating). In contrast, the droplets from the FR composites are non-flaming and self-extinguish before reaching the cotton indicator, thereby allowing all composites to achieve a V-0 rating. This improvement is attributed to the early formation of an expanded carbonaceous char layer, which increases melt viscosity, suppresses flaming drips, and mitigates the hazard of burning droplets falling onto combustible substrates. APP can generate phosphoric acid species that promote dehydration and carbonization, while lignin provides an aromatic carbon source. Meanwhile, non-combustible gases released from APP decomposition, such as NH_3_ and H_2_O, dilute the combustible volatiles and make it harder for the flame to continue after the external ignition source is removed. The LOI results show the same improvement trend. Pure PLA has a low LOI value of 22%, while the LOI values of the composites increase to 32.5% for PLA/Q-gCN, 34.5% for PLA/Q-gCNFS-1.5, 37% for PLA/Q-gCNFS-3, and 34.5% for PLA/Q-gCNFS-6. Among the composites, PLA/Q-gCNFS-3 shows the highest LOI value, indicating the strongest resistance to sustained combustion.

#### 3.3.3. Cone Calorimetry

Cone calorimetry was used to evaluate the fire behavior of bulk materials under continuous external heat flux. This test provides reaction-to-fire information under defined forced-combustion conditions, including heat release rate, total heat release, mass loss, and fire-growth-related parameters. Unlike MCC, Cone calorimetry reflects the combined effects of sample geometry, external heating, char integrity, volatile release, and heat feedback during burning [54]. While MCC highlights intrinsic pyrolytic heat release at the microscale, Cone calorimetry evaluates bulk reaction-to-fire behavior where physical char barrier efficacy dominates. In contrast to the MCC rankings, PLA/Q-gCNFS-3 achieves the best overall flame retardancy under forced flaming conditions (pHRR of 257.1 kW/m^2^ and THR of 52.6 MJ/m^2^). This shift underscores that bulk fire suppression relies on the optimal balance between the 2D g-C_3_N_4_ nanosheet skeleton and 0D FS inorganic crosslinkers (1:3 ratio). At this ideal ratio, fumed silica effectively seals char micro-fissures through dynamic Si–O–P crosslinking without causing particle agglomeration or diluting the intumescent matrix, creating a continuous physical shield that far outweighs intrinsic chemical combustibility alone.

**Figure 4:**
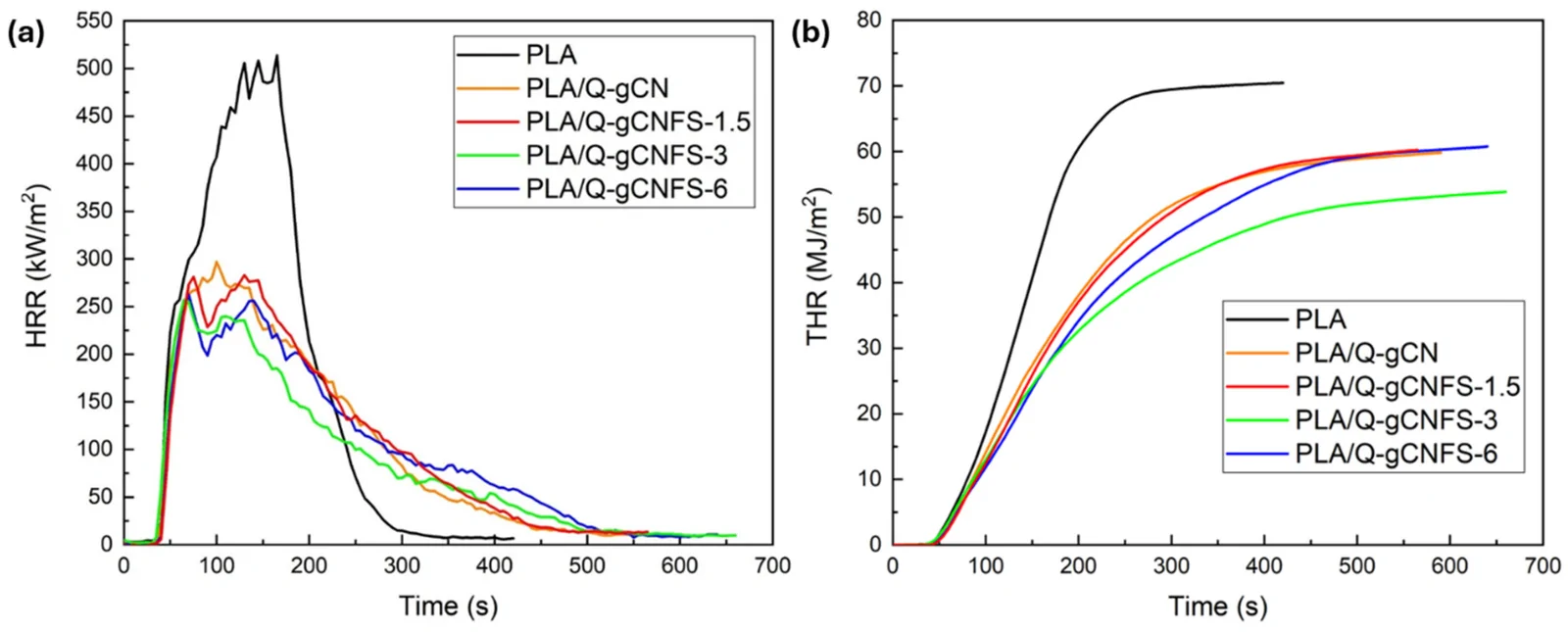
(**a**) HRR, (**b**) THR curves of neat PLA and PLA/FR composites under cone calorimeter tests.

As shown in Table 4, pure PLA shows the most severe combustion behavior, with a pHRR of 513.5 kW/m^2^, a THR of 69.6 MJ/m^2^, and a Maximum Average Rate of Heat Emission (MARHE) of 305.7 kW/m^2^. As shown in Figure 4a, its HRR curve rises sharply after ignition and reaches a high peak within a short time, indicating rapid and concentrated heat release during the early burning stage. After the peak, the heat release drops quickly, showing an intense but short combustion process.

After introducing the flame-retardant system, all composites show clear changes in heat release behavior. The pHRR values decrease to 257–297 kW/m^2^, and the HRR curves become broader and smoother, as shown in Figure 4a. The THR curves in Figure 4b also show that the total heat release is reduced after adding the flame-retardant system. It should be noted that the THR values for PLA/Q-gCN, PLA/Q-gCNFS-1.5, and PLA/Q-gCNFS-6 lie in a very narrow range of 58.7–59.7 MJ/m^2^; given standard experimental variations, these three formulations are considered statistically equivalent in terms of total heat release. The most pronounced drop is achieved by PLA/Q-gCNFS-3, which further lowers the THR to 52.6 MJ/m^2^ (representing a ~24.4% reduction relative to neat PLA). For PLA/Q-gCN, the sharp peak of pure PLA is suppressed into a lower and flatter heat-release plateau. This change indicates that the combustion process is no longer dominated by a short burst of heat release but instead continues at a lower intensity for a longer period. In contrast, the three PLA/Q-gCNFS samples show a two-stage heat-release behavior. The first stage corresponds to a low-intensity and rapid release of volatile products. The second stage appears as a gradually decreasing tail rather than a sharp drop after the peak. This tail suggests that a small amount of fuel is still released from the residue or that the residue is slowly oxidized after the main burning stage. Because the char layer and gCN/FS barrier limit heat transfer and volatile diffusion, this later heat release occurs at a lower intensity and decreases gradually. The MARHE values also decrease after introducing the flame-retardant system. As shown in Table 4, pure PLA shows a high MARHE value of 305.7 kW/m^2^, while all composites show much lower values of 165.3–190.3 kW/m^2^. This decrease means that the composites produce less sustained heat during burning, which reduces heat feedback to the material surface and limits the further release of combustible volatiles. Therefore, the flame is less able to grow and maintain high intensity. Among the composites, PLA/Q-gCNFS-3 shows the lowest MARHE value of 165.3 kW/m^2^, consistent with its lowest pHRR and THR.

The time to reach pHRR also changes significantly. Pure PLA reaches its peak at 165 s, while the composites reach their peaks earlier, in the range of 65–130 s. This indicates that the improved fire performance does not result from delaying peak formation, but from reducing the heat release during burning.

Overall, the Cone calorimetry results show that the flame-retardant system reduces fire intensity and changes the heat-release process from a rapid and concentrated event to a lower-intensity process spread over time.

### 3.4. Flame Retardant Mechanism

#### 3.4.1. Condensed Phase

**Figure 5:**
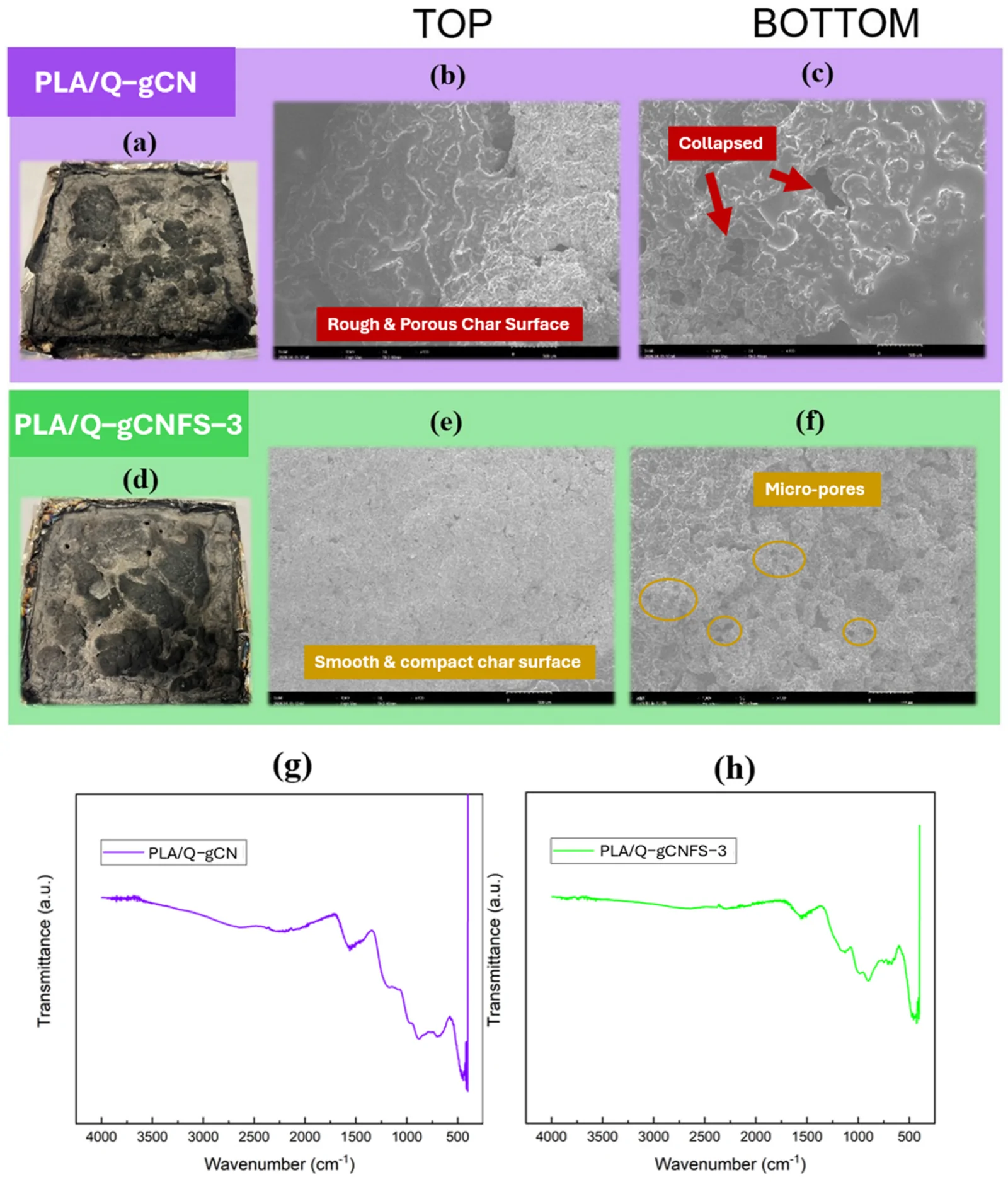
Char residue morphology and FTIR spectra after Cone calorimetry: (**a**–**c**) digital photograph, top-surface SEM image, and bottom-surface SEM image of PLA/Q-gCN; (**d**–**f**) digital photograph, top-surface SEM image, and bottom-surface SEM image of PLA/Q-gCNFS-3 (all SEM images were taken at an accelerating voltage of 10 kV, SE mode, WD = 40 mm, magnification: 100×, scale bar = 500 μm); and (**g**,**h**) FTIR spectra of the corresponding char residues.

To understand the condensed-phase behavior, the post-burn residues were examined by combining the macroscopic cone char images, SEM observations of the top and bottom char surfaces, and FTIR spectra of the cone residues, as shown in Figure 5a–g. The comparison between PLA/Q-gCN and PLA/Q-gCNFS-3 reveals clear differences in residue continuity, pore structure, and solid-phase composition, which help explain the better fire performance of PLA/Q-gCNFS-3.

At the macroscopic level, PLA/Q-gCN shows a nonuniform residue with uneven carbonization, incomplete surface coverage, and several open or depressed regions (Figure 5a). This appearance suggests that the char layer does not develop into a continuous protective barrier over the whole surface. Instead, the residue contains locally thick carbonized patches together with weaker regions that remain open. Such spatial nonuniformity makes heat transfer and volatile release easier through the less protected areas, so the barrier effect is limited even though some local regions retain char. In contrast, PLA/Q-gCNFS-3 shows a more continuous residue surface in the central region (Figure 5d). The surface is largely covered, and the visible defects are small and scattered. This morphology suggests that the expanding char layer can form and consolidate into a more coherent barrier during burning. Small, distributed holes are present, which indicate pressure release through smaller pathways rather than failure through one dominant leakage channel. This kind of residue is favorable for maintaining thermal shielding during prolonged cone exposure.

The SEM results are consistent with macroscopic observations. For PLA/Q-gCN, the top char surface shows strong heterogeneity, with obvious differences in texture and local discontinuities across the flame-exposed side (Figure 5b). The bottom side is also irregular and porous, with openings and locally collapsed regions (Figure 5c). These features are an indication of an uneven structure between the outer skin and inner char, which agrees with the macroscopic coverage seen in Figure 5a. In contrast, PLA/Q-gCNFS-3 exhibits a much more uniform top surface (Figure 5e). The flame-exposed side appears smoother and more compact, with fewer obvious discontinuities. The bottom side also shows a finer and more homogeneous porous morphology, without one large opening dominating the structure (Figure 5f). This means that the residue of PLA/Q-gCNFS-3 can maintain surface continuity while still accommodating gas release through more evenly distributed pores. Such a structure is important because it lowers the chance that gas flow and stress become concentrated in one location. As a result, the char is less likely to fracture into a large crack or open channel. It is worth noting that the structural integrity of the post-combustion char layer is intrinsically linked to the dispersion state and spatial distribution of the nano-additives within the initial polymer matrix. In unburned nanocomposites, severe filler agglomeration often induces localized stress concentration and compositional heterogeneity, which inevitably triggers macro-crack formation and mechanical failure during rapid intumescent char expansion. Conversely, the continuous, smooth, and defect-free char surface observed in PLA/Q-gCNFS-3 indirectly implies that the ethanol-assisted pre-assembly strategy successfully suppresses 0D FS self-aggregation and 2D g-C_3_N_4_ restacking, promoting a uniform nanoscale dispersion of the hybrid network throughout the PLA matrix.

The residue FTIR spectra further support this difference in condensed-phase quality (Figure 5g,h). Both samples show a band near ~1600 cm^−1^, which is assigned to C=C vibration and indicates the presence of carbonized and aromatized structures after burning. Both samples also show broad absorptions in the 1200–900 cm^−1^ region, which are related to phosphate-containing structures such as P=O, P–O–P, and P–O–C formed from APP during combustion. These signals indicate that APP participates in condensed-phase reactions and helps build a phosphate-rich char structure. However, PLA/Q-gCNFS-3 shows stronger and clearer features in the silica-related regions than PLA/Q-gCN. In particular, the Si–O–Si bands near ~1100 cm^−1^, ~800 cm^−1^, and ~450–500 cm^−1^ are more evident in PLA/Q-gCNFS-3, confirming that silica remains in the cone residue. Hence, the char of PLA/Q-gCNFS-3 is not only phosphate-rich and carbonized, but also reinforced by an inorganic silica framework.

Overall, the results in Figure 5 show that the improved fire behavior of PLA/Q-gCNFS-3 is closely related to a stronger condensed-phase barrier effect. The residue is not only chemically more stable, but also physically more continuous and less permeable. This difference is clearly reflected in the macroscopic char appearance, the SEM morphology, and the residue FTIR signatures. Although direct nanoscale spatial mapping of the unburned composites via cross-sectional TEM or SEM/EDS was not performed in the present work, the uniform char reinforcement, consistent FTIR blue-shifts, and high reproducibility in Cone calorimetry metrics collectively confirm the effective dispersion of the pre-assembled hybrid. Direct cross-sectional TEM and SEM/EDS characterizations of unburned cryogenic fracture surfaces will be systematically pursued in future work to further quantify the interfacial spatial distribution.

#### 3.4.2. Gas Phase

**Figure 6:**
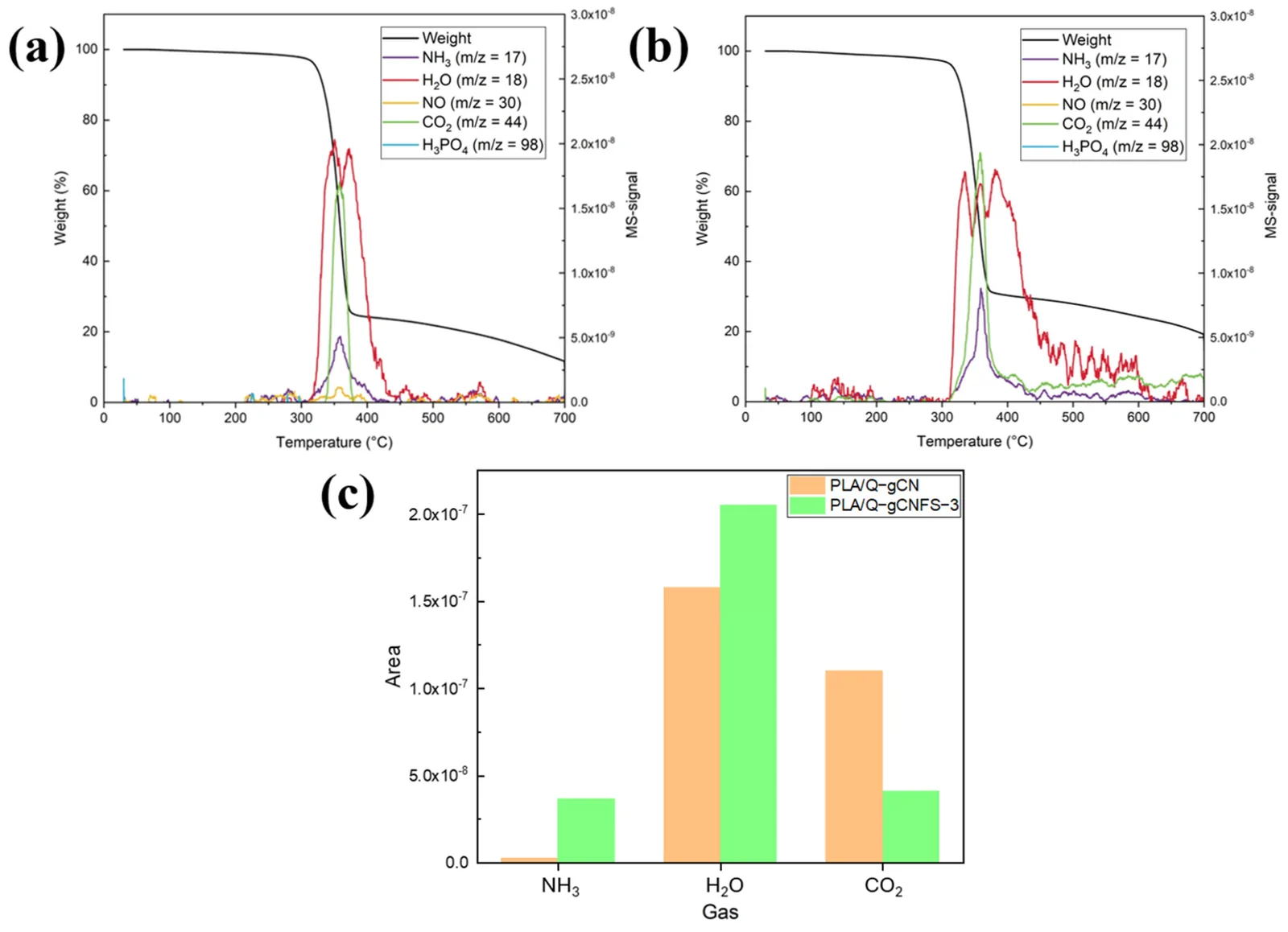
TG–MS profiles of (**a**) PLA/Q-gCN (**b**) PLA/Q-gCNFS-3 (**c**) Integrated areas of NH_3_, H_2_O, and CO_2_ signals for PLA/Q-gCN and PLA/Q-gCNFS-3.

Figure 6 compares the gaseous products released during pyrolysis for PLA/Q-gCN and PLA/Q-gCNFS-3 by TG-MS. For both samples, the main gas evolution occurs in the major decomposition region at about 330–390 °C, which matches the sharp mass-loss step in the TG curves, as shown in Figure 6a. A similar main gas-evolution region is also observed for PLA/Q-gCNFS-3, as shown in Figure 6b. Among the monitored species, H_2_O and CO_2_ are the main volatile products, while NH_3_ is also detected at lower intensity. This result indicates that pyrolysis in both systems involves dehydration, decomposition of oxygen-containing structures, and the release of nonflammable nitrogen-containing species associated with the APP-containing flame-retardant pathway.

Clear differences appear when PLA/Q-gCNFS-3 is compared with PLA/Q-gCN. As shown in Figure 6c, PLA/Q-gCNFS-3 exhibits larger integrated signal areas at *m*/*z* = 17 and *m*/*z* = 18, while the integrated area for CO_2_ (*m*/*z* = 44) is reduced. It is important to clarify that TG-MS serves as a qualitative and comparative tool for evolved species rather than a strict quantitative mass balance. Furthermore, the ion signal at *m*/*z* = 17 is a combined envelope containing both NH_3_^+^ and the characteristic OH^+^ fragment generated by electron ionization of H_2_O (*m*/*z* = 18). Because both formulations contain an identical 15 wt% APP loading and pristine g-C_3_N_4_ remains thermally stable up to ~550 °C (well above the main degradation window of 330–390 °C), APP is the sole primary source of initial nitrogenous volatiles. Therefore, the higher intensity observed at *m*/*z* = 17 in PLA/Q-gCNFS-3 is largely driven by enhanced catalytic dehydration (H_2_O release and its corresponding fragmentation) alongside APP decomposition, rather than an unexplainable increase in pure NH_3_ emission.

Taken together, the TG-MS results in Figure 6a–c show that PLA/Q-gCNFS-3 changes the pyrolysis pathway in two connected ways. In the gas phase, it promotes the release of more nonflammable species, especially NH_3_ and H_2_O. In the solid phase, it reduces carbon loss as CO_2_ and supports greater carbon retention in the residue. This gas-phase behavior is consistent with the lower heat release and better overall flame-retardant performance of PLA/Q-gCNFS-3 observed in MCC and Cone calorimetry.

#### 3.4.3. Proposed Mechanism

**Figure 7:**
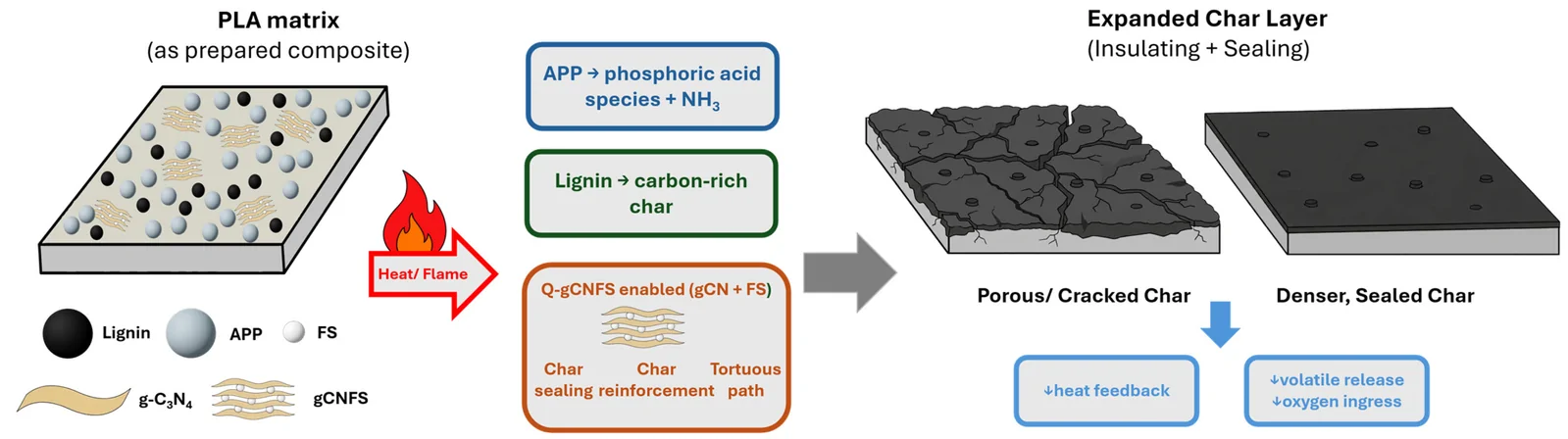
Proposed flame-retardant mechanism of PLA/APP/lignin/Q-gCNFS composites.

Figure 7 illustrates the proposed flame-retardant mechanism of the combined APP/lignin/Q-gCNFS system. Under heat or flame exposure, APP decomposes to form phosphoric acid species and nonflammable gases such as NH_3_. The phosphoric acid species promote dehydration and carbonization of PLA degradation products and lignin, while lignin provides an aromatic carbon-rich source for char formation. At the same time, the pre-assembled Q-gCNFS hybrid strengthens the developing residue. Specifically, 2D gCN nanosheets construct a stable skeletal framework, while the sub-1 wt% FS-derived silica nanoparticles act as high-temperature inorganic crosslinkers and vitreous network modifiers rather than bulk physical gap-fillers. During pyrolysis, the highly dispersed silica nanoparticles chemically react at elevated temperatures with APP-derived polyphosphoric acid species to construct thermally stable Si–O–P crosslinked structures and a silicate-rich glassy coating. This dynamic inorganic crosslinking increases the melt viscosity of the char surface, effectively sealing micro-fissures and suppressing crack propagation during char expansion, thereby yielding a highly continuous, dense, and tortuous protective barrier. As a result, heat transfer, oxygen ingress, and the escape of combustible volatiles are restricted through a more tortuous pathway. Meanwhile, the release of NH_3_ and H_2_O dilutes the combustible gases near the flame and helps expand the forming char. Therefore, the improved flame retardancy of PLA/Q-gCNFS mainly comes from the combined action of APP-induced dehydration and gas dilution, lignin-assisted carbon formation, and Q-gCNFS-reinforced char sealing.

## 4. Conclusions

This study demonstrates that pre-assembled gCNFS powders enhance the flame-retardant performance of the PLA/APP/lignin system through a coupled condensed-phase and gas-phase mechanism. Among the tested formulations, PLA/Q-gCNFS-3 shows the best overall fire performance in Cone calorimetry, with pHRR, THR, and MARHE reduced by approximately 50.0%, 24.4%, and 45.9%, respectively, compared with pure PLA. Post-burn analyses further show that this formulation forms a more continuous, denser, and less permeable residue, as supported by the macroscopic char morphology, SEM observations, and residue FTIR features associated with phosphate-containing structures and retained Si–O–Si frameworks. These results indicate that APP and lignin promote the formation of an expanded carbonaceous char, while the pre-assembled gCNFS hybrid improves char sealing and reinforces the residue framework. At the same time, TG–MS suggests a more sustained release of nonflammable species at elevated temperatures, which contributes to gas-phase dilution during burning. Together, these effects reduce heat feedback, suppress volatile release and oxygen ingress, and lead to improved and more stable flame-retardant behavior of PLA/Q-gCNFS-3. These findings provide a simple pre-assembly strategy for improving the flame retardancy of PLA-based composites before melt processing. This strategy may support the development of safer biodegradable polymer composites for applications that require both environmental compatibility and fire safety, such as packaging, 3D-printed components, electronic housing, and transportation-related parts.

## Figures and Tables

**Table 1:** Formulations and absolute compositions of PLA and flame-retardant composites.

Sample ID	PLA (wt%)	APP (wt%)	Lignin (wt%)	Q-Additive Type	g-C_3_N_4_ (wt%) ^a^	FS (wt%) ^a^	FS: g-C_3_N_4_ Ratio
PLA	100	-	-	-	0	0	-
PLA/Q-gCN	75	15	7	g-C_3_N_4_	3.0	0	-
PLA/Q-gCNFS-1.5	75	15	7	gCNFS hybrid	1.8	1.2	1: 1.5
PLA/Q-gCNFS-3	75	15	7	gCNFS hybrid	2.25	0.75	1: 3
PLA/Q-gCNFS-6	75	15	7	gCNFS hybrid	2.57	0.43	1: 6

Note: The prefix “Q” denotes the Quaternary system, which comprises the primary ternary flame-retardant matrix (75 wt% PLA, 15 wt% APP, and 7 wt% Lignin) combined with 3.0 wt% of the functional quaternary additive (pristine g-C_3_N_4_ or pre-assembled gCNFS hybrids). The listed g-C_3_N_4_ and FS wt% values represent their final absolute concentrations relative to the total mass of the bulk composite material.

**Table 2:** MCC test of PLA and PLA/FR composites.

Samples	Char Yield (%)	THR (kJ/g)	pHRR (W/g)	T_max_ (°C)
PLA	1.7 ± 0.9	16.5 ± 0.8	449.0 ± 12.1	392.4 ± 0.3
PLA/Q-gCN	15.3 ± 0.0	11.5 ± 0.4	366.1 ± 26.6	387.6 ± 0.1
PLA/Q-gCNFS-1.5	15.7 ± 0.5	14.2 ± 0.3	379.2 ± 1.7	385.7 ± 1.8
PLA/Q-gCNFS-3	17.2 ± 1.0	12.6 ± 0.4	334.1 ± 20.8	384.3 ± 0.4
PLA/Q-gCNFS-6	18.2 ± 1.0	12.0 ± 0.1	324.0 ± 0.1	387.3 ± 0.3

**Table 3:** UL-94 ratings and LOI values of neat PLA and PLA/FR composites.

Samples	Dripping	Cotton Ignition	Ave. t1 (s)	Ave. t2 (s)	UL-94	LOI (%)
PLA	Y	Y	11.5	14	V-2	22.0 ± 0.5%
PLA/Q-gCN	Y	N	0.4	0.0	V-0	32.5 ± 0.5%
PLA/Q-gCNFS-1.5	Y	N	1.2	0.5	V-0	34.5 ± 0.5%
PLA/Q-gCNFS-3	Y	N	0.0	0.0	V-0	37.0 ± 0.5%
PLA/Q-gCNFS-6	Y	N	0.0	0.0	V-0	34.5 ± 0.5%

Note: Ave. t1 and Ave. t2 represent the average after-flame times obtained from five repeated measurements. UL-94 V-0 classification requires individual after-flame times t1, t2 ≤ 10 s, total after-flame time ≤ 50 s, and no flaming drips that ignite surgical cotton. V-2 classification allows flaming drips that ignite cotton indicator, provided individual t_1_, t_2_ ≤ 30 s.

**Table 4:** Data of Neat PLA and PLA/FR Composites under Cone Calorimeter Tests.

Sample	pHRR (kW/m^2^)	THR (MJ/m^2^)	MARHE (kW/m^2^)	Time to pHRR (s)
PLA	513.5	69.6	305.7	165
PLA/Q-gCN	296.9	58.7	190.3	100
PLA/Q-gCNFS-1.5	282.9	58.9	185.4	130
PLA/Q-gCNFS-3	257.1	52.6	165.3	65
PLA/Q-gCNFS-6	262.6	59.7	171.1	70

## Data Availability

The original contributions presented in this study are included in the article/Supplementary Material. Further inquiries can be directed to the corresponding author.

## Supplementary Materials

The following supporting information can be downloaded at: https://www.mdpi.com/article/10.3390/polym18212622/s1, Table S1: MCC data of PLA and PLA/FR composites.

## References

[1] Balla E., Daniilidis V., Karlioti G., Kalamas T., Stefanidou M., Bikiaris N.D., Vlachopoulos A., Koumentakou I., Bikiaris D.N. (2021). Poly(Lactic Acid): A Versatile Biobased Polymer for the Future with Multifunctional Properties-from Monomer Synthesis, Polymerization Techniques and Molecular Weight Increase to PLA Applications. Polymers.

[2] Reshma C.S., Remya S., Bindu J. (2024). A Review of Exploring the Synthesis, Properties, and Diverse Applications of Poly Lactic Acid with a Focus on Food Packaging Application. Int. J. Biol. Macromol..

[3] Rajendran D.S., Venkataraman S., Jha S.K., Chakrabarty D., Kumar V.V. (2024). A Review on Bio-Based Polymer Polylactic Acid Potential on Sustainable Food Packaging. Food Sci. Biotechnol..

[4] Joseph T.M., Kallingal A., Suresh A.M., Mahapatra D.K., Hasanin M.S., Haponiuk J., Thomas S. (2023). 3D Printing of Polylactic Acid: Recent Advances and Opportunities. Int. J. Adv. Manuf. Technol..

[5] Alhulaybi Z.A. (2023). Fabrication and Characterization of Poly(Lactic Acid)-Based Biopolymer for Surgical Sutures. ChemEngineering.

[6] Chen T., Wen Y., Zhu D., Feng W., Song B., Wang Q. (2025). Comparison of Bioabsorbable Screw versus Metallic Screw Fixation for Tibial Tubercle Fractures in Adolescents: A Retrospective Cohort Study. BMC Musculoskelet. Disord..

[7] Yang Z., Yin G., Sun S., Xu P. (2024). Medical Applications and Prospects of Polylactic Acid Materials. iScience.

[8] Giammaria V., Capretti M., Del Bianco G., Boria S., Santulli C. (2024). Application of Poly(Lactic Acid) Composites in the Automotive Sector: A Critical Review. Polymers.

[9] Gomes D.A., Cusioli L.F., Mantovani D., Tusset A.M., Parmegiani Marcucci S.M., Lenzi G.G., Mapossa A.B., Bergamasco R. (2025). Three-Dimensional Prototyping with PLA for the Electrical Sector: Perspectives and Challenges in Material Development. Energies.

[10] Senila L., Kovacs E., Resz M.A., Senila M., Becze A., Roman C. (2024). Life Cycle Assessment (LCA) of Bioplastics Production from Lignocellulosic Waste (Study Case: PLA and PHB). Polymers.

[11] Rezvani Ghomi E., Khosravi F., Saedi Ardahaei A., Dai Y., Neisiany R.E., Foroughi F., Wu M., Das O., Ramakrishna S. (2021). The Life Cycle Assessment for Polylactic Acid (PLA) to Make It a Low-Carbon Material. Polymers.

[12] Lors C., Leleux P., Park C.H. (2024). State of the Art on Biodegradability of Bio-Based Plastics Containing Polylactic Acid. Front. Mater..

[13] Fogašová M., Figalla S., Danišová L., Medlenová E., Hlaváčiková S., Vanovčanová Z., Omaníková L., Baco A., Horváth V., Mikolajová M. (2022). PLA/PHB-Based Materials Fully Biodegradable under Both Industrial and Home-Composting Conditions. Polymers.

[14] Khouri N.G., Bahú J.O., Blanco-Llamero C., Severino P., Concha V.O.C., Souto E.B. (2024). Polylactic Acid (PLA): Properties, Synthesis, and Biomedical Applications—A Review of the Literature. J. Mol. Struct..

[15] Quan Y., Zhang W., Marquez J.A.D., Guo L., Tanchak R., Shen R., Wang Q. (2025). Sustainable Biomass-Based Flame Retardants: Recent Advances in Starch, Phytic Acid, and Chitosan Systems for Polymeric Materials. Emerg. Manag. Sci. Technol..

[16] Shen R., Quan Y., Zhang Z., Ma R., Wang Q. (2022). Metal-Organic Framework as an Efficient Synergist for Intumescent Flame Retardants against Highly Flammable Polypropylene. Ind. Eng. Chem. Res..

[17] Vasile C., Baican M. (2023). Lignins as Promising Renewable Biopolymers and Bioactive Compounds for High-Performance Materials. Polymers.

[18] Zhang X., Wang Z., Ding S., Wang Z., Xie H. (2024). Fabrication of Flame-Retardant Ammonium Polyphosphate Modified Phytic Acid-Based Rigid Polyurethane Foam with Enhanced Mechanical Properties. Polymers.

[19] Zhang Y., Liu L., Yao M., Feng J., Xue Y., Annamalai P.K., Chevali V., Dinh T., Fang Z., Liu H. (2024). Functionalizing Lignin by in Situ Solid-Phase Grafting Ammonium Polyphosphate for Enhancing Thermal, Flame-Retardant, Mechanical, and UV-Resistant Properties of Polylactic Acid. Chem. Eng. J..

[20] Peil S., Mouhoubi R., Streekstra R., Ridard H., Veith L., Ali W., Mayer-Gall T., Duvigneau J., Wurm F.R. (2024). Encapsulation of Ammonium Polyphosphate in Lignin Nanocontainers Enhances Dispersion and Flame Retardancy in Polylactic Acid Foams. ACS Appl. Polym. Mater..

[21] Zhang G., Wang J., Dai Y., Liu M., Hu Y., Shang Q., Hu L., Meng Z. (2025). Novel Lignin-Based Ionic Liquid with APP toward Fire Safety PLA with Well-Balanced Comprehensive Performance. Ind. Crops Prod..

[22] Sun Y., Liu M., Zhang Z., Liu H., Shi D., Ying J., Mu W., Li D., Kong I. (2025). Effect of Silane-Modified Ammonium Polyphosphate on the Mechanical, Thermal, and Flame-Retardant Properties of Rice Husk/Polylactic Acid Composites. J. Compos. Sci..

[23] Won S., Jung M., Bang J., Cho S.Y., Choi I.G., Kwak H.W. (2024). Lignin-Based Flame Retardant via Sequential Purification-Nanoparticle Formation, and N[Sbnd]P Coupled Chemical Modification. Int. J. Biol. Macromol..

[24] Li Y., Li L., Bo C., Hu L., Yang X., Zhou Y. (2025). An Intrinsic Lignin-Based Flame Retardant for Phenolic Foams: Synthesis and Fire Safety Enhancement. React. Funct. Polym..

[25] Zhu S., Jiang Z., Liu Z., Zheng Y., Ge B., Ma M., Chen S., He H., Shi Y., Zhu Y. (2025). Construction of Environmentally Friendly Multifunctional Core-Shell Flame Retardant and Its Application in Flame Retardant Polylactic Acid. Int. J. Biol. Macromol..

[26] Yoo H., Jo J., Kim S.J., Koo B. (2025). Development of Silylated Lignin-Based Intumescent Flame Retardants for Biodegradable Plastics. Polymers.

[27] Puglia D., Santulli C., Sarasini F. (2022). Micro and Nanolignin in Aqueous Dispersions and Polymers.

[28] Zheng S., Li W., Chen Y., Yang H., Cai Y., Wang Q., Wei Q. (2024). Synergistic Effect of Stereo-Complexation and Interfacial Compatibility in Ammonium Polyphosphate Grafted Polylactic Acid Fibers for Simultaneously Improved Toughness and Flame Retardancy. Int. J. Biol. Macromol..

[29] Cayla A., Rault F., Giraud S., Salaün F., Fierro V., Celzard A. (2016). PLA with Intumescent System Containing Lignin and Ammonium Polyphosphate for Flame Retardant Textile. Polymers.

[30] Laoutid F., Vahabi H., Saeb M.R., Dubois P. (2021). Lignin as a Flame Retardant for Biopolymers. Micro and Nanolignin in Aqueous Dispersions and Polymers: Interactions, Properties, and Applications.

[31] Réti C., Casetta M., Duquesne S., Bourbigot S., Delobel R. (2008). Flammability Properties of Intumescent PLA Starch and Lignin. Polym. Adv. Technol..

[32] Marquez J.A.D., Zhang W., Suphavilai N., Shetty M., Jeong H.K., Wang Q. (2025). Enhancing the Inherent Flame Retardancy of Polylactic Acid by Anchoring Phytic Acid-Lysine Using Epoxidized Tannic Acid. ACS Appl. Polym. Mater..

[33] Cao X., Chi X., Deng X., Liu T., Yu B., Wang B., Yuen A.C.Y., Wu W., Li R.K.Y. (2020). Synergistic Effect of Flame Retardants and Graphitic Carbon Nitride on Flame Retardancy of Polylactide Composites. Polym. Adv. Technol..

[34] Zhang W., Wu W., Meng W., Xie W., Cui Y., Xu J., Qu H. (2020). Core-Shell Graphitic Carbon Nitride/Zinc Phytate as a Novel Effcient Flame Retardant for Fire Safety and Smoke Suppression in Epoxy Resin. Polymers.

[35] Xu W., Chen R., Xu J., Zhong D., Cheng Z. (2020). Nickel Hydroxide and Zinc Hydroxystannate Dual Modified Graphite Carbon Nitride for the Flame Retardancy and Smoke Suppression of Epoxy Resin. Polym. Degrad. Stab..

[36] Singh N.B., Usman U.L., Abdala A. (2025). Nanomaterials in Fire Protection: Innovations for Enhanced Safety and Durability. Nano-Struct. Nano-Objects.

[37] Shi Y., Xing W., Wang B., Hong N., Zhu Y., Wang C., Gui Z., Yuen R.K.K., Hu Y. (2016). Synergistic Effect of Graphitic Carbon Nitride and Ammonium Polyphosphate for Enhanced Thermal and Flame Retardant Properties of Polystyrene. Mater. Chem. Phys..

[38] Xu Z., Chu F., Wang C., Wang X., Hou Y., Hu W., Song L. (2024). Fabrication of a 2D/2D g-C3N4@ZnTDA Nanosheets Catalyst for Highly Efficient Degradation of 2,4-Dibromophenol and Improved Flame Retardancy of Polyurethane Foam. Sustain. Mater. Technol..

[39] Gaddam S.K., Pothu R., Boddula R. (2020). Graphitic Carbon Nitride (g-C3N4) Reinforced Polymer Nanocomposite Systems—A Review. Polym. Compos..

[40] Okoli U., Rishi K., Beaucage G., Kammler H.K., McGlasson A., Chauby M., Narayanan V., Grammens J., Kuppa V.K. (2023). Dispersion of Modified Fumed Silica in Elastomeric Nanocomposites. Polymer.

[41] Ko J.W. (2024). Graphitic Carbon Nitride as Reinforcement of Photopolymer Resin for 3D Printing. Polymers.

[42] Hasannezhad A., Amirpoor S., Dolati A., Sharifi R. (2024). Boosting the Efficiency of Water-Based Intumescent Coatings with Fumed Silica Nanoparticles: A Synergistic Approach. Mater. Today Commun..

[43] Wang Y., Liu B., Chen R., Wang Y., Han Z., Wang C., Weng L. (2023). Synergistic Effect of Nano-Silica and Intumescent Flame Retardant on the Fire Reaction Properties of Polypropylene Composites. Materials.

[44] Marquez J.A.D., Quan Y., Zhu X., Sue H.J., Wang Q. (2024). Predicting the Processability of Polymers in a Twin-Screw Extruder: CFD Simulation and Experimental Validation. Ind. Eng. Chem. Res..

[45] Zhang R., Xiao X., Tai Q., Huang H., Yang J., Hu Y. (2012). Preparation of Lignin-Silica Hybrids and Its Application in Intumescent Flame-Retardant Poly(Lactic Acid) System. High Perform. Polym..

[46] Ren J., Li Y., Lin Q., Li Z., Zhang G. (2022). Development of Biomaterials Based on Plasticized Polylactic Acid and Tea Polyphenols for Active-Packaging Application. Int. J. Biol. Macromol..

[47] Niu D., Yu W., Yang W., Xu P., Liu T., Wang Z., Yan X., Ma P. (2023). Flame-Retardant and UV-Shielding Poly (Lactic Acid) Composites with Preserved Mechanical Properties by Incorporating Cyclophosphazene Derivative and Phosphorus-Modified Lignin. Chem. Eng. J..

[48] Carrasco F., Pérez O.S., Maspoch M.L. (2021). Kinetics of the Thermal Degradation of Poly(Lactic Acid) and Polyamide Bioblends. Polymers.

[49] Cayla A., Rault F., Giraud S., Salaün F., Sonnier R., Dumazert L. (2019). Influence of Ammonium Polyphosphate/Lignin Ratio on Thermal and Fire Behavior of Biobased Thermoplastic: The Case of Polyamide 11. Materials.

[50] Li F., Lv W., Huang D., Zeng C., Wang R. (2023). Physicochemical Properties, Thermal Stability, and Pyrolysis Behavior of Antioxidative Lignin from Water Chestnut Shell Obtained with Ternary Deep Eutectic Solvents. Molecules.

[51] Rawool S.A., Samanta A., Ajithkumar T.G., Kar Y., Polshettiwar V. (2020). Photocatalytic Hydrogen Generation and CO2 Conversion Using G-C3N4 Decorated Dendritic Fibrous Nanosilica: Role of Interfaces between Silica and g-C3N4. ACS Appl. Energy Mater..

[52] Yu Y., Wang J., Yan Z., Jing Q., Liu P., Xu B. (2022). Supramolecular Precursor Strategy to Construct G-C3N4/Silica Hybrid Nanosheets for Photocatalytic Degradation of Dye and Antibiotic Pollutants. Nanomaterials.

[53] Shen J., Liang J., Lin X., Lin H., Yu J., Wang S. (2022). The Flame-Retardant Mechanisms and Preparation of Polymer Composites and Their Potential Application in Construction Engineering. Polymers.

[54] Quan Y., Zhang Z., Tanchak R.N., Wang Q. (2022). A Review on Cone Calorimeter for Assessment of Flame-Retarded Polymer Composites. J. Therm. Anal. Calorim..

