# Supplementary material for: G-C3N4-Orchestrated Multi-Component Synergy for Enhanced Flame Retardancy in PLA
Source: Polymers (Basel). 2026 Oct 23;18(21):2622. doi: 10.3390/polym18212622 (PMC13765845; doi:10.3390/polym18212622)
Supplement: Supplementary file 1 [file polymers-18-02622-s001.zip › polymers-4583794-supplementary.pdf]

Supplemental material

Table S1. MCC data of PLA and PLA/FR composites

Sample	Char Yield (%)	THR (kJ/g)	pHRR (W/g)	T_{max} (°C)
PLA	1.7	16.5	449.0	392.4
PLA_5%APP	9.8	16.2	463.9	387.0
PLA_15%APP	9.4	14.0	445.0	385.9
PLA/0.1gCN	-	15.9	429.1	393.8
PLA/1gCN	-	15.1	421.4	397.4
PLA/5gCN	-	16.0	456.0	396.5
PLA_1%Lignin	-	18.0	444.9	385.6
PLA_5%Lignin	-	16.6	417.8	359.5
PLA_10%Lignin	-	13.5	355.7	327.0
PLA_7%Lignin_3%gCN	12.4	14.3	349.9	355.9
PLA_15%APP_3%gCN	8.8	13.7	441.4	387.1
PLA_15%APP_7%Lignin	10.5	12.9	415.3	387.3
